# Species interactions and eco-evolutionary dynamics of dispersal: the diversity dependence of dispersal

**DOI:** 10.1098/rstb.2023.0125

**Published:** 2024-08-12

**Authors:** Dries Bonte, Sally Keith, Emanuel A. Fronhofer

**Affiliations:** ^1^ Department of Biology, Ghent University, K.L. Ledeganckstraat 35, Gent B-9000, Belgium; ^2^ Lancaster Environment Centre, Lancaster University, Lancaster LA1 4YQ, UK; ^3^ ISEM, University of Montpellier, CNRS, IRD, EPHE, Montpellier 34095, France

**Keywords:** eco-evolutionary dynamics, interactions, dispersal

## Abstract

Dispersal plays a pivotal role in the eco-evolutionary dynamics of spatially structured populations, communities and ecosystems. As an individual-based trait, dispersal is subject to both plasticity and evolution. Its dependence on conditions and context is well understood within single-species metapopulations. However, species do not exist in isolation; they interact locally through various horizontal and vertical interactions. While the significance of species interactions is recognized for species coexistence and food web functioning, our understanding of their influence on regional dynamics, such as their impact on spatial dynamics in metacommunities and meta-food webs, remains limited. Building upon insights from behavioural and community ecology, we aim to elucidate biodiversity as both a driver and an outcome of connectivity. By synthesizing conceptual, theoretical and empirical contributions from global experts in the field, we seek to explore how a more mechanistic understanding of diversity–dispersal relationships influences the distribution of species in spatially and temporally changing environments. Our findings highlight the importance of explicitly considering interspecific interactions as drivers of dispersal, thus reshaping our understanding of fundamental dynamics including species coexistence and the emergent dynamics of metacommunities and meta-ecosystems. We envision that this initiative will pave the way for advanced forecasting approaches to understanding biodiversity dynamics under the pressures of global change.

This article is part of the theme issue ‘Diversity-dependence of dispersal: interspecific interactions determine spatial dynamics’.

## 1. Introduction

Dispersal, birth and death rates play pivotal roles in shaping life histories [1] and governing population dynamics of species residing in spatially structured environments. These distinct populations are commonly framed as patches within a metapopulation [2–4]. When accounting for multiple interacting or non-interacting species, their dynamics are studied as metacommunities [5,6]. Scaling up in time and space, dispersal, alongside speciation and extinction, constitutes one of the three fundamental processes governing the diversity and distribution of life on Earth [7]. Dispersal and behaviour become especially relevant when humans alter landscapes [8,9]. The spatial distribution of species diversity, whether local, regional or global, arises because of local population dynamics and dispersal across various scales. Dispersal, operating at these scales, is intricately regulated by both emigration and immigration rates. These rates are influenced by landscape characteristics such as distances between patches and the permeability of the matrix, environmental conditions such as weather and climate, and intrinsic species traits like size, dispersal morphology, perceptual ability and physiology [10,11]. These factors collectively influence connectivity among populations and communities, thereby mediating gene flow, demographic patterns and species interactions [7,12].

Biotic interactions, including competition, facilitation and predation, play pivotal roles in shaping individual performance and, by extension, density regulation within populations [13,14]. The recognition of the importance of species interactions as fundamental drivers of species coexistence is well-established [15–17]. However, dispersal has primarily been studied from individual or population perspectives, often overlooking how its dynamics are intertwined with interspecific interactions within biodiverse environments. Given that species inhabit complex ecological networks, interspecific interactions likely mediate the plasticity and evolution of dispersal strategies [18–20]. These interactions manifest at local scales but are influenced by processes that shape community structure at broader scales across both time and space, such as ecological selection, mass effects and ecological drift. As a consequence, dispersal is also a key linker between ecology and evolution [21,22].

The distribution and abundance of species across landscapes are consequently governed by four fundamental processes: (i) environmental filtering based on local abiotic conditions; (ii) biotic interactions arising from competition and trophic dynamics; (iii) dispersal among habitat patches; and (iv) ecological drift resulting from demographic stochasticity [6,23]. These processes underpin the foundational pillars—neutral and patch dynamics, species sorting and mass effects—of the metacommunity framework [5,24], contingent upon the contributions of dispersal limitation and environmental heterogeneity to the spatial organization of biodiversity. At broader temporal scales, the processes of speciation introduce an additional dimension to the formation of spatial biodiversity patterns. Subsequently, integrating the magnitudes and directions of abiotic resource fluxes situates ecosystems within such a meta-framework [25]. Recognizing the profound behavioural components underlying interaction and dispersal processes, the incorporation of behavioural ecology [26], as well as the environmental determinants of these behaviours spanning micro- to macroscales [27], emerges as a critical next step towards advancing our understanding of biodiversity organization across multiple spatial scales.

The metacommunity framework offers a valuable lens through which to understand the mediation of dispersal by biotic interactions and the resulting impacts on feedback loops between local and regional pools of species diversity. Currently, this framework predominantly serves as a variance partitioning tool, elucidating the presumed significance of spatial and environmental drivers in community assembly [28–31], including those influencing microbiomes [32]. With the integration of contemporary network [33–35] and coexistence [31,36] theories in spatial ecology, a more mathematically formalized approach has facilitated a deeper mechanistic comprehension of the multifaceted roles played by both horizontal (such as competition and mutualism) and vertical (including predator–prey dynamics and host–parasite interactions) interactions. This formalization also constitutes a primary focal point within this special issue, aiming to unravel the intricate interplay of dispersal–diversity or dispersal–interaction feedbacks (see Box 1).

Box 1: **Formulating density dependence.**

In a simple Lotka–Volterra formulation (see also [6,37]), the dynamics of *n* interacting species with abundance *N*_*i*_ of species *i* in a specific location (patch, *x*) depend on their intrinsic rate of increase $r_{0,i}$ and the community matrix that captures all intra- and interspecific interactions $\alpha_{i,j}$ :

(1.1) $$\frac{dN_{i,x}}{dt}=\left(r_{0,i}-\sum_{j}^{n}\alpha_{i,j}N_{j,x}-m_{i}+\sum_{l}\frac{\left(1-\mu \right)m_{i}N_{i,l}}{c_{l}}\right)N_{i,x},$$

where $m_{i}$ represents the species-specific emigration rate, μ as the dispersal costs [38,39], *l* as the patches adjacent to *x* and $c_{l}$ is the number of connections leaving the patch *i*. Seeing dispersal as a reaction norm [40,41], under density-dependent emigration, we assume that

(1.2) $$m_{i}=f\left(N_{i,x}\right),$$

where (equation 1.2) is a nonlinear function of *N,* e.g. as derived in [41]. Note that the exact shape is not relevant here. We here argue that, besides impacting the evolution of dispersal [42,43], interspecific interactions should also be taken into account when thinking about dispersal plasticity [44]:

(1.3) $$m_{i}=f\left(N_{i,x},N_{j,x}\right),$$

which makes dispersal depend on both the intraspecific and interspecific densities. The exact weighting function must be derived, but, for a horizontal competitive metacommunity a simple sum of the competition coefficient weighted densities as for density regulation ( $\sum_{j}^{n}\alpha_{i,j}N_{j,x}$ ) should be the adequate solution. In the case of vertical interactions, like predator–prey and host–parasite, the reaction norm may be multidimensional (see [18] for a meta-experiment).

Dispersal is not merely a passive phenomenon; rather, it represents an active component of an organism’s life history, from protists to plants and animals [45]. Departure, transfer and settlement during dispersal are strongly influenced by individual conditions and behaviour [44]. Thus, dispersal is a crucial, most often plastic, trait with significant implications for the organization of biodiversity at meso- and macroevolutionary scales. The specific modes of dispersal are contingent upon the utilization of environmental information, including the acquisition of resources (e.g. food and mating partners) and information on resource availability or accessibility [44]. This information may stem from abiotic cues reflecting environmental quality and future fitness prospects, as well as from biotic processes within food webs or interactions such as competition [18,46].

Despite its significance, metacommunity theory has traditionally assumed dispersal to be a fixed, landscape-determined parameter, neglecting its context-dependent nature as a life-history trait [6]. This discrepancy between the framing and methodologies of population and behavioural ecology, on one hand, and community ecology and biogeography, on the other, has hindered efforts to explicitly link dispersal with biotic interactions. Recognizing the importance of intraspecific density and interspecific diversity dependence as population regulatory mechanisms through dispersal is critical, as they influence reproduction, survival and ultimately, species coexistence.

**Figure 1.**
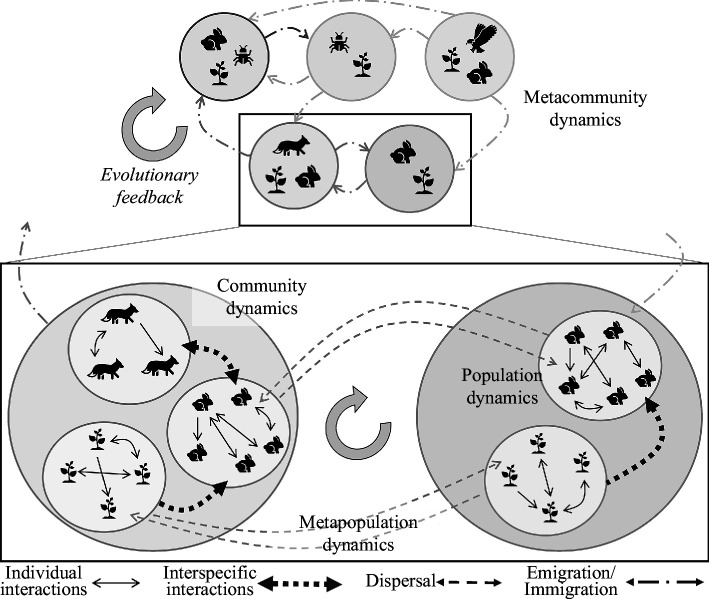
Conceptual figure demonstrating how population dynamics (birth, death processes from individual interactions), species interactions (functional responses, competition strengths as a function of the species densities) and patch interactions (individual dispersal emerging into species’ emigration and immigration rates) determine the organization of metacommunities. Interactions within and among species drive dispersal, which feeds back on these population and community dynamics. Metacommunity dynamics thus integrate single-species spatial population dynamics, which are in turn governed by individual interactions. Different symbols represent different species, full dashed circles collect the interacting species within the patch. Eco-evolutionary feedbacks on dispersal prevail at the level of population, metapopulation and metacommunity dynamics.

The population sizes of species, in conjunction with their identities and the environmental mediators that influence them, collectively dictate the local fitness prospects and thereby their persistence within intricate interaction networks. Rooted in the realms of single-species ecology and evolution, it is well-established that fitness prospects within spatially structured systems are heavily contingent upon dispersal as a density-dependent and density-regulating mechanism. Consequently, both species emigration and immigration rates are hypothesized to be contingent upon the local community or interaction network structure while also serving as major structuring processes. Dispersal thus interweaves intricately with dynamic shifts in both local and regional community assembly, from both ecological and evolutionary standpoints (see figure 1; [21,47]).

This collection of papers aims to delve deeper into three key aspects:

### (a) Characterizing diversity-dependent dispersal

The first set of papers explores the mechanisms underlying diversity-dependent dispersal. Specifically, these papers investigate how and under what conditions diversity-dependent dispersal patterns arise in response to interspecies interactions.

As an initial step towards understanding the significance of interspecific interactions on dispersal, Bestion *et al*. [48] conducted a comprehensive horizon scan of existing evidence in the field, followed by a meta-analytical synthesis. Their findings reveal that detrimental interactions and complex communities tend to enhance the dispersal of the receiver, whereas beneficial ones tend to suppress its dispersal. Moreover, the magnitude of this response is inversely correlated with the strength of species interactions and is contingent upon the overall composition of the community. Fundamental principles derived from optimality models offer valuable insights into how anticipated changes in fitness influence the plasticity of dispersal in simple ecological communities.

Thierry *et al.* [49] find that although dispersal rates of ciliate microcosms are dependent on temperature, density and the presence of heterospecifics (or alternative strains), these factors did not interact with one another, i.e. thermal dependency of dispersal was not altered by species interactions.

Beyond well-studied predatory, mutualistic and competitive interactions, parasites and their hosts often engage in intricate spatial relationships, wherein the movement of one organism directly affects the other. Through the development of a metapopulation model, Baines & Shaw [50] demonstrate that host dispersal rates may produce varying responses—increasing, decreasing or causing non-monotonic changes in parasite prevalence—depending on the specific type of parasite-dependent dispersal (PDD). Through experimental investigations, Regimbal & Baines [51] further illustrate the intricate nature of negative PDD, which can lead to complex cascading effects on host demography by constraining dispersal and subsequently heightening the host’s susceptibility to cannibalism. Furthermore, given that multiple parasites may infect hosts simultaneously, they become subject to interspecific interactions for host resources.

Using spider mites as a model system, Godinho *et al.* [52] demonstrate that virulence and transmission—two crucial components of infection dynamics—can be independently influenced under conditions of coinfection, resulting in increased transmission (e.g. disease dispersal) without concurrent changes in virulence. Such interactions among parasites may have significant implications for epidemiology and the evolution of parasite traits, albeit without necessarily shaping the virulence–transmission trade-off.

### (b) Impact on community assembly in metacommunities and the structure of food webs

Another focal point is to elucidate how diversity-dependent dispersal influences community assembly processes within metacommunities and meta-food webs. By examining these dynamics, we can uncover the intricate ways in which dispersal patterns shaped by species diversity affect the composition and structure of ecological communities across interconnected habitats.

Insights garnered from pairwise interactions underscore the profound influence of diversity- or interaction-dependent dispersal on eco-evolutionary dynamics within spatially organized food webs and communities. Within all metacommunities, analysing dispersal strategies provides valuable insights into how the spatial arrangement of patches acts as a filter, influencing species composition through ecological selection on dispersal traits. Khattar *et al*. [53 have successfully replicated empirical dispersal patterns across multiple scales with a simulation model, offering comprehensive insights into the factors influencing the success and diversity of dispersal strategies in various ecological contexts. Their findings reveal that the success of unique, context-specific dispersal strategies in metacommunities can be shaped by the interaction between intra- and interspecific competition and spatiotemporal variability in habitat conditions. Similarly, Fajgenblat *et al*. [54] employed a simulation-based approach to investigate the concurrent evolution of dispersal and local adaptation. Their findings highlight that the evolution of dispersal can significantly influence evolution-mediated priority effects, suggesting that evolutionary characteristics of foundation species influence the subsequent colonization or persistence of other species. This process was particularly prominent in species-poor communities undergoing rapid change.

Variation in dispersal among species not only influences diversity patterns within metacommunities of competing species but also plays a crucial role in mutualistic interactions, particularly in seed dispersal and the spread of plant species. Carlo *et al*. [55] reveal that frugivory interactions exhibit predominantly negative density-dependent (NDD) effects in both temperate and tropical bird–plant communities across North and South America. They find that birds exhibit stronger preferences for rare fruit resources while underutilizing dominant ones. Such NDD dynamics in frugivore–plant interactions serve as robust equalizing mechanisms for the dispersal processes of fleshy-fruited plant species across temperate and tropical ecosystems, thereby shaping and maintaining diversity patterns. Moreover, in cases where mutualistic interactions involve strong reciprocal selection, coevolutionary hotspots can significantly influence the richness, dispersal, extinction and persistence patterns of mutualistic species. Cosmo *et al*. [56], adopting a connectivity perspective, demonstrate that coevolutionary hotspots promote colonization in spatial networks, enabling species to expand their distribution across landscapes even amid changing environmental conditions.

Current studies examining the properties of food webs and emerging spatial patterns often oversimplify dispersal as a fixed, non-flexible connectedness property. McPeek *et al*. [57] utilize computer simulations and analytical models to illustrate that dispersal strategies employed by interacting species profoundly impact the extent of mass effects in meta-food webs. Additionally, different dispersal rules have varied effects on the emergence of spatial patterns in population abundances. Lawton *et al*. [58] further demonstrate that pattern formation critically depends on dispersal rules utilized by predators and prey, as well as the complexity of the network. Understanding meta-food web patterns in realistic landscapes, such as the species–area relation, necessitates simultaneous consideration of landscape configuration and local food web dynamics (Ryser *et al*. [59]).

### (c) Scaling to macroecology and biogeography

Lastly, the collection addresses how insights gained from studying diversity-dependent dispersal at local and regional scales can be extrapolated to understand broader patterns in macroecology and biogeography. By scaling up the analyses, the papers aim to uncover the implications of diversity-dependent dispersal for larger-scale biodiversity patterns and distribution dynamics across landscapes and ecosystems.

Transitioning from regional metacommunities to macroecological scales introduces new challenges, particularly in upscaling local and regional processes to appropriate spatiotemporal dimensions, including species formation processes (Alzate & Hagen [60]). Challenges of scaling up range from disciplinary issues such as consensus on the language used around dispersal, to more methodological issues that include transmutation, changes in the dominance of different processes and emergent properties [27]. Hagen *et al*. [61] subsequently employ a dynamic archipelago biodiversity model to illustrate how landscape properties, dispersal and species interactions shape macroecological biodiversity patterns. Specifically, they demonstrate how competition can enhance allopatric speciation and highlight the emergence of multiple intermediate dispersal–diversity relationships under varying model conditions.

## 2. Moving forward

To conclude this special issue, Fronhofer *et al*. [62] synthesize principles derived from single-species population dynamics, enabling their transposition to a multispecies context. Despite the increased complexity of diverse communities, fundamental principles concerning fitness expectations in both space and time remain applicable to most biotic interactions. The review identifies novel and analogous principles within metacommunities and meta-food webs, emphasizing the challenges posed by significantly increased trait dimensionality in complex communities. Additionally, it underscores the crucial role of co-dispersal as a key modulator of biodiversity within species networks [63]. This special issue represents a pivotal initial step towards unravelling the tight interplay between diversity and dispersal in ecological and evolutionary realms. The concluding article outlines remaining open questions and avenues for further research in the field of biodiversity studies.

We hope this special issue inspires the readers to reflect on the role of dispersal, its plasticity and evolution into the organization of biodiversity at local, regional and global spatial scales as well as in contemporary and deep time. The collection of papers demonstrates the diversity dependence of dispersal, and its recurrent impact on diversity both theoretically and across a range of empirical systems. Yet, to identify how far we can apply general principles to the interplay of dispersal and species interactions across metacommunities, we need to broaden our exploration to marine biomes [64], which are noticeably lacking in this issue—we were unable to identify researchers working on such questions within this realm.

While marine megafauna might experience species interactions and dispersal dynamics analogous to terrestrial systems, many marine animals have a dispersive larval phase with wide variation in swimming capacity from essentially passive particles at the whim of ocean currents (e.g. coral) to capable swimmers (e.g. fishes), potentially increasing the importance of long-distance dispersal relative to terrestrial systems [65]. Dispersal via larvae is perhaps less likely to be influenced directly by species interactions because offspring are produced en masse and interactions at that stage are largely trophic-based. However, on evolutionary timeframes, species interactions are an important driver of dispersal dynamics. One of the clearest examples is that of coral mass spawning, which is an adaptation to reduce predation pressure on individual larvae [66]. The three-dimensional structure of the oceans can also lead to vertical dispersal, which can be related to ontogeny or on shorter timescales allowing access to different hydrodynamic regimes altering dispersal potential [67]. Therefore, we have substantial opportunities to learn by comparing and contrasting across biomes, including freshwater, which straddles the dynamics of both marine and terrestrial systems [68,69].

We also note that there are currently too few empirical tests of underlying principles identified through theoretical models, which may well reflect logistical difficulties around capturing the intricacies of both multiple ecological communities and their spatially dependent interconnections (for a recent discussion of dispersal experimental evolution see [70]). Therefore, it is imperative that as a research community, we search for solutions that will enable large-scale empirical research through initiatives such as distributed experiments. Recently, methods available to track species movements have experienced rapid development, which has the potential to create new opportunities and bring traditionally unobservable marine dispersal into focus.

However, one of the most important questions remains unanswered: *does diversity-dependence of dispersal matter for predicting responses of biodiversity under global change?* While some of us are convinced (E.A.F.), others believe its importance will be context- and scale-dependent (S.K.) and others (D.B.) remain more sceptical on its importance relative to the unprecedented socio-ecological and socio-economical changes we are witnessing today. Bringing the theory to action remains one of the pressing challenges for ecologists across the world.

## Data Availability

This article has no additional data.
